# New Multi-Keyword Ciphertext Search Method for Sensor Network Cloud Platforms

**DOI:** 10.3390/s18093047

**Published:** 2018-09-12

**Authors:** Lixia Xie, Ziying Wang, Yue Wang, Hongyu Yang, Jiyong Zhang

**Affiliations:** 1 School of Computer Science and Technology, Civil Aviation University of China, Tianjin 300300, China; zywang_yjs16@cauc.edu.cn (Z.W.); ywang2_yjs15@cauc.edu.cn (Y.W.); hyyang@cauc.edu.cn (H.Y.); 2 School of Computer and Communication Science, Swiss Federal Institute of Technology in Lausanne, CH-1015 Lausanne, Switzerland; zhangjiyong@gmail.com

**Keywords:** sensor network, cloud, multi-keyword, ciphertext search, KNN, the top-k result, privacy protection

## Abstract

This paper proposed a multi-keyword ciphertext search, based on an improved-quality hierarchical clustering (MCS-IQHC) method. MCS-IQHC is a novel technique, which is tailored to work with encrypted data. It has improved search accuracy and can self-adapt when performing multi-keyword ciphertext searches on privacy-protected sensor network cloud platforms. Document vectors are first generated by combining the term frequency-inverse document frequency (TF-IDF) weight factor and the vector space model (VSM). The improved quality hierarchical clustering (IQHC) algorithm then generates document vectors, document indices, and cluster indices, which are encrypted via the k-nearest neighbor algorithm (KNN). MCS-IQHC then returns the top-k search result. A series of experiments proved that the proposed method had better searching efficiency and accuracy in high-privacy sensor cloud network environments, compared to other state-of-the-art methods.

## 1. Introduction

Recent advancements in sensor networks, big data, and cloud computing technologies have placed increasingly stringent requirements on the transfer and outsourcing of sensor data to cloud servers [1,2]. In a cloud environment, a user has on-demand data access. However, first, the sensor data owner and sensor meta uploader need to effectively transfer data via the server’s network. To resolve the resulting contradiction between the confidentiality and functionality of the data stored in the cloud server, Wang had proposed the concept of a ciphertext search technology in Reference [3]. Cloud servers are semi-credible; therefore, privacy protection is a crucial aspect of their functionality; and thus, a ciphertext search method is currently the search method of choice most commonly deployed in cloud environments. Traditional ciphertext search algorithms for sensor data are generally inefficient. They do not perfectly target multiple keywords or effectively protect sensor data in the cloud. There is currently an urgent demand for new, high-efficiency, and multi-keyword ciphertext search techniques.

Multi-keyword searching, with fuzzy matching keywords and public-key encryption, was first proposed by Qin et al. [4]. It is an effective search technique but is inefficient and time-consuming. Cao et al. [5] proposed a multi-keyword ranked search algorithm (MRSA) for encrypted cloud data (ECD), which used the results of coordinate matching to measure the similarity between a keyword and document, whilst the similarity of the inner product was used to evaluate the coordinate matching results. A document index was represented by a Boolean quantization, so documents with the same number of keywords had the same correlation score; thus, the search accuracy was relatively low. Wang [6] established a technique that sorted document keywords based on term frequency-inverse document frequency (TF-IDF). Though this approach reduced search time, it required specific encryption search software, which increased the computational complexity of the system. Handa et al. [7] set-up offline document clusters based on the similarities between document keywords. These clusters were then used to create a clustering index. Whilst creative, this method does not allow the user to adjust the keyword values as desired, which leads to an overall lack of adaptability in practice. Chen et al. [8] proposed a multi-keyword ranked search over encrypted data based on a hierarchical clustering index (MRSE-HCI), but this method required a special extension when constructing the index dimension, which is associated with a high computational (calculation) cost. Li et al. [9] proposed a verifiable cloud encryption keyword search method that supported multi-user access. By using heavy encryption technology, the multi-user ciphertext search was implemented, the user’s query permission was updated dynamically, and signature-binding keyword indices were used. Its associated encrypted files, enabled fast access to data in a cloud environment. Lu et al. [10] proposed a multi-keyword search method based on a min-hash function. After the known search content, the server uses the TF-IDF method to compare the search request with the searchable index and returns the first n-most relevant results. This method requires two servers to perform ciphertext search during ciphertext search, which increases the communication cost of the user.

In summary, research into developing multi-keyword ciphertext search methods for cloud storage has achieved some results, but search efficiency, accuracy, and adaptability still need to be further improved. Therefore, more in-depth research is needed to address these issues. Thus, the major contribution of our work will be:

- We propose a method for building document vectors, by combing term frequency-inverse document frequency (TF-IDF) with Vector Space Model (VSM) to optimize search efficiency, and we propose an improved quality hierarchical clustering (IQHC) algorithm based on the quality hierarchical clustering algorithm (QHC).
- We incorporated this improved algorithm into a new multi-keyword ranked search method for encrypted sensor data and used the new search method to implement a multi-keyword ciphertext search over encrypted cloud data, based on the improved quality hierarchical clustering algorithm (MCS-IQHC).
- We ran a series of experiments to test the quality of MCS-IQHC, MRSE, and MRSE-HCI methods, and to demonstrate that the proposed method facilitates highly-efficient and accurate multi-keyword ciphertext searching, under the simulated sensor cloud network environment.

This paper is organized as follows. Section 2 discusses related work on ciphertext search technology analysis. In Section 3, the research background of our proposed method is illustrated. In Section 4, we first detail the improved algorithm, then the new multi-keyword ciphertext search method is described. The proposed experiment and traditional methods are compared and analyzed in Section 5. Section 6 summarizes the conclusion, and discusses future work

## 2. Related Work

This section mainly analyzes the existing work on ciphertext search technology, including the QHC algorithm, TF-IDF, and VSM.

### 2.1. Quality Hierarchial Clustering Algorithm Analysis

Several quality hierarchical clustering (QHC) [4] algorithms were implemented in cloud storage systems to improve traditional K-means [11] clustering algorithms. These QHC methods were based on clustering results that used hierarchical strategies and limited the number of samples according to cluster size. A ciphertext search that established hierarchical clustering indices, shortened the cipher text search time and improved the search efficiency, whilst meeting the dynamical requirements of dynamically changing data in the cloud storage environment. However, this algorithm had some shortcomings, such as high-dimensional file vectors, which have little contribution to the document search or ciphertext searches than can be directly ignored. Thus, the algorithm will result in redundant computation, low search efficiency, and long search time.

### 2.2. TF-IDF and VSM

TF-IDF is a statistical approach commonly used for information retrieval and data mining. It can measure the importance of a keyword to a document (or a document collection). “TF” refers to the frequency of keywords in a single document, while IDF is the inverse document frequency, i.e., the frequency of a keyword in an entire document collection. IDF can measure the universal importance of keywords. TF-IDF can determine the value of each keyword, then uses this value to identify the needed documents. In this study, we selected these keywords as the features and the product of TF and IDF as the keyword value. When building a document vector, the number of appearances and the significance of each keyword in the document collection were included in the clustering process.

The Vector Space Model (VSM) is a text representation method, commonly used in information retrieval applications. If the document is composed of multiple irrelevant characteristics, the features can be words, terms, phrases, or other elements of the document; thus, there is no sequence between each character. VSM assigns a value to each feature, sets its value as a coordinate, then converts your document into a vector in space [12]. All documents are vectorized to transform the text-processing problem into a mathematical operation among vectors. Mathematical calculations are among the many vector characteristics (e.g., distance, angle), which allow for accurate measurement of the similarity among document contents. In this study, we used the vector distance as a measurement of similarity that is applied to VSM, to enhance the effects of clustering. These results are discussed in detail below and they resulted in a strong search accuracy when combining TF-IDF and VSM to sort the ciphertext search results.

## 3. Background

### 3.1. System Model

**Figure 1:**
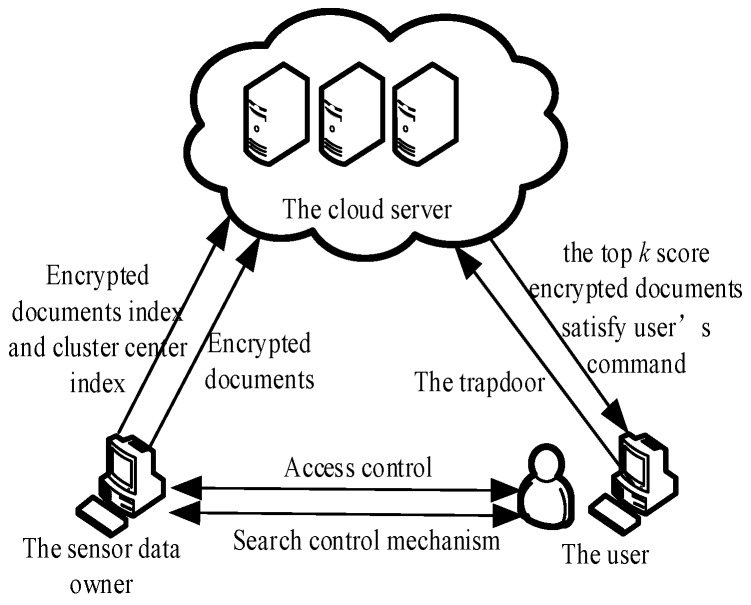
System Model.

The MRSE-IQHC ciphertext search method proposed in this paper involves three entities: Sensor data owners, users, and cloud servers. These three entities and the data stream together form a system model, in which the sensor data owner and user are honest and trustworthy, whilst the cloud server is semi-credible. Figure 1 shows a flowchart of the system model.

In a sensor network cloud, the sensor data owner is the “entity” of the documents, from which it extracts the keywords. The proposed method can be used to generate a cluster index and document index through the document vectors, then upload the encrypted index and encrypted documents to the cloud server.

The user’s identity is now highly credible because the identities are authenticated. The user is the entity that performs the search operation. During the search phase, the user can define the values of keywords and create search requests. It then generates an encrypted search vector (i.e., a trapdoor), which is assisted by the sensor data owner. After uploading the trapdoor to the cloud server, the user waits for the cloud server to return the search results.

A single cloud server [13], is a semi-trusted server that stores encrypted documents and encrypted indexes. The cloud server can execute a search operation based on the trapdoor sent by the user, then the desired search results become available to the user.

### 3.2. Security Threat Model

The system may be attacked from outside of the sensor network cloud [14], inside the cloud, or via a collusion attack from inside or outside the cloud [15]. Attacks from outside the sensor network cloud are usually network attacks. Attackers must break through the cloud server’s security system to steal user data from within. Attacks from outside the sensor network cloud are not discussed in this paper.

Collusion attacks [16] from the inside and outside, may severely threaten sensor networks and sensor data. In a collusion attack or an inside attack, the attacker seeks to obtain plaintext information from other sensor data. This type of attack seriously harms the system and may cause leakage of sensitive sensor data. The attacker can intercept communications between the sensor data owner, user, and cloud server to derive additional information from the intercepted information. The search process falls into two security threat models, as defined by the information stolen by an attacker.

In the Known Ciphertext model, the attacker has the sensor data owner’s encrypted document, encrypted document index, and cluster index. The attacker also has the user’s encrypted search vector input.

In the Known Background Knowledge model, the attacker knows more statistical information about the sensor data set, such as the total number of keywords, keyword frequency, data correspondence, and trapdoor settings.

## 4. Multi-Keyword Ciphertext Search Method

By analyzing the sensor data stream between the user, data owner, and cloud server, we designed an improved clustering algorithm and multi-keyword ciphertext search method, which can be easily implemented in an existing cloud storage environment. In this section, we first describe the improved algorithm steps. Next, we propose a new ciphertext search method based on the improved algorithm. Finally, we analyze the security of the new method based on the security threat model.

### 4.1. Improved Quality Hierarchical Clustering Algorithm

The ciphertext search algorithm MRSE-HCI [6], builds vector documents solely based on the existence of keywords and does not consider keyword frequency or keyword significance. The search results only include documents that contain keywords. This is because the algorithm cannot sort the outcomes in accordance with other meaningful aspects of the keywords within the corpus before returning them to the user; this results in a significant deviation between the search results and the user’s expectations. The proposed method was designed to resolve any problems by combining TF-IDF with VSM to build document vectors.

There are several distinct problems associated with the low efficiency and high computational cost of multi-keyword ciphertext searches in sensor network cloud storage environments. The problems include high-dimensional results, significant redundancy among document vector dimensions, and sparse data distribution. The proposed algorithm combines principal component analysis (PCA) [17] with quality hierarchical clustering (QHC) [6], to minimize the dimensions of document vectors in the vector space, whilst retaining vital characteristics, before re-clustering the data. The steps to the proposed algorithm are as follows.

1. Generate *n* samples of the document vectors ***G*_1_**, ***G*_2_**, …, ***G_n_***, ***G_i_*** = (***g***_1_, ***g***_2_, …, ***g_p_***)^T^, (*i* = 1, 2, ..., *n*) through TF-IDF and VSM. These vectors all have *p* dimensions. Standardize the vectors. When *n* > *p*, construct a sample matrix. Apply the following transformations to the sample elements:
  (1) $$z_{ij}=\frac{g_{ij}-\bar{d_{j}}}{s_{j}},i=1,2,\cdots ,n;j=1,2,\cdots ,p$$
  where $\bar{d_{j}}$ is the expectation; *s_j_* is the standard deviation.
2. Obtain a normalized sample matrix ***M*** as follows:
  (2) $$\bar{d_{j}}=\frac{\sum_{i=1}^{n}g_{ij}}{n},s_{j}=\sqrt{\frac{{\sum_{i=1}^{n}\left(g_{ij}-\bar{d_{j}}\right)}^{2}}{n-1}},j=1,2,\cdots ,p$$
  Determine ***M***’s covariance matrix ***C,*** where ***M^T^*** is the transpose matrix of ***M***.
  (3) $$C=\frac{M^{T}M}{n-1}$$
3. Apply the Singular Value Decomposition method to solve the characteristic equation (|***C***-*λ**I_p_***| = 0) of the Sample Covariance Matrix ***M***. Then, obtain *p* characteristic roots and sort them in descending order. Use contribution rate *η* from Formula (4), to confirm the number of principal components and mark this value as *m*. Choose a value for *η*, the original information is represented by the *m* principal components. Use each latent root *λ_j_* (*j* = 1, 2, …, *m*), determined by solving
  ***R**b* = *λ_j_b*,
  to obtain *m* unit eigenvectors *b_j_* (*j* = 1, 2, …, *m*).
  (4) $$\sum_{j=1}^{m}\lambda_{j}/\sum_{j=1}^{p}\lambda_{j}\geq \eta$$
4. Convert the normalized data variables into principal components. ***P*_1_** is the first principal component, ***P*_2_** is the second principal component, ..., ***P_m_*** is the *m*th principal component. Obtain dimension reduction vectors after replacing the document vectors with the new principal components.
  (5) $$P_{l}=z_{l}{}^{T}b_{j}^{},j=1,2,\ldots ,m;l=1,2,\ldots ,m$$
5. Set the maximum number of documents in each cluster (denoted as *TH*), and the minimum threshold value of the correlation value marked as min(***S***). The Euclidean distance is used to measure the correlation between the documents, and to establish a relationship between each document and cluster center.
6. Cluster the document vectors, after a dimension reduction, via the K-means algorithm [18]. *k* is the number of the clusters. When *k* is unstable, we add a new cluster center and generate *k* + 1 new random cluster centers to the clusters. Then, we compare the minimum threshold min(***S***) of the correlation value with the minimum correlation score min(***S_t_***), (*t* = 1, 2, ..., *k*) in each cluster. If min(***S_t_***) < min(***S***), we add a new clustering center and re-cluster. We repeat this process until *k* is stable, then proceed with Step 7.
7. We consider the clustering center set in Step 6 as the first-level cluster center ***C*_0_**, and verify the number of documents in each cluster sequentially. If the number of documents exceeds the pre-set threshold value TH, we divide the cluster into multiple sub-clusters; otherwise, do not divide. Regard any newly divided sub-clusters as a second-level cluster center, and repeat Step 7 until the number of documents in all clusters falls under threshold TH.
8. Repeat steps 1–7 until all clusters satisfy the cluster dependency and quantity requirements. The clustering process is complete.

### 4.2. MCS-IQHC

This paper proposed MCS-IQHC based on the IQHC method. Document vectors are first constructed via VSM and TF-IDF. Later, IQHC is applied to cluster the vectors and reduce their dimensions. The k-nearest neighbor (KNN) [19] query algorithm is then used to encrypt the index and search vectors. A search request, encrypted by the value of a user-defined keyword is established, whilst the ciphertext search is performed. In this section, we describe the implementation steps of the new method and its security. The MCS-IQHC process is then implemented in a typical cloud storage environment as follows.

1. Document vector execution. The sensor data owner considers the product of TF and IDF as the value of keywords. In VSM, a vector represents the documents in the upload queue. The value at that position, denotes each dimension of the document vectors. Then, a dictionary ***D_w_*** and the document vector set ***D*** are created.
2. Index creation. The IQHC clusters the document vectors and generates a *u*-bit dictionary ***D_w._*** This is implemented by the sensor data owner. The clustering results are used to build a cluster and document index. The length of the document-index and cluster-index vectors are *u* bits.
3. Index encryption. The sensor data owner generates a *u*-bit random vector ***S*** = {0,1}*^u^* and two *u* × *u* reversible matrices ***Z*_1_**, ***Z*_2_** as the key, using the KNN [17] query algorithm at random. Each bit of the division vector ***S*** is a random variable 0 or 1. Thus, there is approximately the same number of 0 and 1 values in ***S***. Each bit in the matrix ***Z*_1_** or ***Z*_2_** is a random integer. Segmentation vector ***S,*** serves as a split indicator for segmenting the document index and cluster index. The sensor data owner divides the cluster index vector ***V*** into two vectors ***V’*** and ***V”*** based on ***S***. This process is random. The *i*th bit of vectors ***V’*** and ***V”*** are denoted by ***V_i_’*** and ***V_i_”***, (*i* = 1, 2, ..., *u*). The transposed matrices ***Z*_1_^T^**, ***Z*_2_^T^** are reversible matrices of ***Z*_1_**, ***Z*_2_**, where ***Z*_1_^T^**, ***Z*_2_^T^** is used to encrypt ***V’*** and ***V”***. When the *i*th term in ***S*** is 0, then ***V_i_”*** = ***V_i_’*** = ***V_i_***, (*i* = 1, 2, ..., *u*); or the *i*th in ***S*** is 1 then ***V_i_’*** = ***V_i_*** − ***V_i_”***, (*i* = 1, 2, ..., *u*); the cluster index is encrypted as ***I_c_*** = {***Z*_1_^T^*V’***, ***Z*_2_^T^*V”***} as is the document index ***I_d_***.
4. Document encryption. The sensor data owner chooses a secure symmetric encryption algorithm to encrypt the documents, then sends the encrypted document set ***D_e_*** with the encrypted document index ***I_d_*** and the encrypted cluster index ***I_c_*** to the cloud server.
5. Trapdoor generation. The user selects keywords, assigns different values to these keywords on demand, and requests that the cloud server return the first k documents that satisfy the demand. The search request is subsequently constructed and sent to the data owner. After receiving the request, the sensor data owner assigns a value to the requested keyword location, according to the dictionary ***D_w_’***, then produces a *u*-bit search vector ***Q*** and encrypts ***Q*** with the matrix ***Z*_1_**^−1^, ***Z*_2_**^−1^. The search vector ***Q*** is then randomly divided into two vectors ***Q’*** and ***Q”*** (based on ***S)*** by the data owner. The *i*th bit in ***Q’*** and ***Q”*** is denoted as ***Q_i_’*** and ***Q_i_”***, (*i* = 1, 2, ..., *u*). If the *i*th bit in ***S*** is 0, then ***Q_i_’*** = ***Q_i_*** − ***Q_i_”*** (*i* = 1, 2, ..., *u*); otherwise the *i*th in ***S*** is 1. This results in the relationship ***Q_i_”*** = ***Q_i_’*** = ***Q_i_***, (*i* = 1, 2, ..., *u*).
  The search request ***Q*** is split into ***Q’*** and ***Q”***; where ***Q’*** and ***Q”*** are encrypted by the matrices ***Z*_1_**^−1^, ***Z*_2_**^−1^ to obtain the trapdoor ***T_d_*** = {***Z*_1_^−1^*Q’***, ***Z*_2_^−1^*Q”***}. The sensor data owner then sends ***T_d_*** to the user.
6. Search process. The user sends the trapdoor ***T_d_*** to the cloud server. The correlation score which marked as *Score* [6] is calculated according to the inner product calculated by the cloud server. Formula (6) shows that the inner product of ***I_c_*** and ***T_d_*** is equal to the inner product of ***V*** and ***Q***. The result of the ciphertext state search is the same as the plaintext state search; the encryption does not affect the accuracy of the search results.
  (6) $$\begin{array}{ll} Score & =I_{c}\cdot T_{w}\\ & =\left\{Z_{1}^{T}V^{\prime },Z_{2}^{T}V^{\prime \prime }\right\}\cdot \left\{Z_{1}^{-1}Q^{\prime },Z_{2}^{-1}Q^{\prime \prime }\right\}\\ & =V^{\prime }Q^{\prime }+V^{\prime \prime }Q^{\prime \prime }\\ & =VQ \end{array}$$
  The cloud server first calculates the inner product of ***T_d_*** and the first-level cluster center in ***I_c_***, then it finds the highest-scoring first-level cluster center. The second-stage cluster center with the highest score is determined by calculating the inner product of ***T_d_*** and the previously obtained sub-cluster center of the first-level cluster center, until reaching the final high-score cluster center. Finally, the inner product of ***T_d_*** and ***I_d_*** are used to obtain the top *k*-scoring encrypted documents and return them to the user.
7. Decryption process. The user sends a decryption request to the sensor data owner, then decrypts the document after receiving the decryption key from the owner.

### 4.3. Security Analysis for MCS-IQHC

In a sensor network cloud storage environment, an attacker may intercept communication data (including sensor data) from any of the three entities involved. Trappable information includes the encrypted clustered index ***I_c_***, the encrypted document index Id, the encrypted document set ***D_e_***, the trapdoor ***T_d_***, and the correlation score *Score*.

In MRSE-IQHC, the cluster index ***I_c_***, document index ***I_d_***, and trapdoor ***T_d_*** are sent to the cloud server, after encryption via KNN. In the encryption stage, each index vector and trapdoor is randomly split into two vectors. Search requests with the same keyword generate different trapdoors, so that the attacker cannot derive the original vectors or plaintext information from them. The sensor data owner encrypts the documents via a symmetric encryption algorithm before uploading it. The encrypted operation satisfies privacy protection requirements [20]. The sensor data owner retains the key, so that the attacker cannot obtain the documents in plaintext. The index and the document are encrypted differently, which provides strong anti-attack capabilities to the search method. The attacker cannot obtain keyword information based only on the correlation score found via the search process, which protects keyword privacy as well. The proposed method effectively guarantees the security of retrieved data under both, the Known Ciphertext Model and the Known Background Knowledge Model.

## 5. Experimental Results and Analysis

### 5.1. Experimental Data and Environmental Configuration

We used the Chinese Corpus of Fudan University [21], for a text clustering and performance test. The corpus contains 9804 documents that span 20 categories including art, history, energy, electronics, and transportation. The diverse data provided a reasonable approximation of real sensor conditions, and thus more practical experimental results, compared to data from a single category.

The experiment configuration used a computer system with an Intel Core i3-2350M CPU @ 2.30 GHz, with 4.0GB RAM. The code (based on the algorithm) was written in Python on a Windows 7 64-bit operating system, which was capable of running TensorFlow to simulate a cloud environment. We classified each document in the training sets of this corpus. Then, we tested MCS-IQHC, MRSE, and MRSE-HCI in the same experimental environment for comparison. With the data sets and experimental environment settings, we conducted our experiments in an environment resembling a real sensor cloud network environment.

### 5.2. Experimental Search Efficiency and Accuracy

We randomly selected documents from the corpus to test the various method search times, and accuracies under different sensor data conditions. We then analyzed the security of each MCS-IQHC result, separately.

#### 5.2.1. Document Quantity Effects on Search Time and Accuracy

We divided the experimental data into five groups. Each group had 150, 300, 450, 600, and 750 documents. Every selected document varied within 1 KB to 50 KB. The dictionary size was ***D_w_*** = 9000, the search request consisted of 10 keywords, and each keyword was assigned a value from 1 to 3. The user requested the return of 10 documents in each iteration of the experiment. We tested the search time and search accuracy in three measurements, based on a variety of document sets of differing sizes.

**Figure 2:**
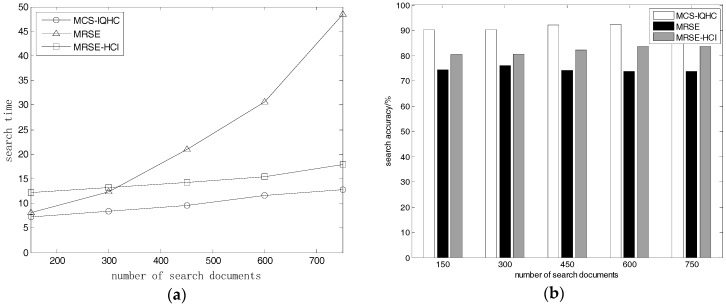
(**a**) Relationship between document quantity and search time of various data sets; (**b**) Relationship between document quantity and search accuracy of various data sets.

As shown in Figure 2a, the search time of MCS-IQHC is close to that of MRSE in certain cases. It is consistently much lower than MRSE-HCI. The search times for MRSE-HCI and MCS-IQHC increased linearly as the document set size increased, whilst the MRSE search time grew exponentially. In Figure 2b, the accuracy of MCS-IQHC is much higher than the other two methods with the increase of the number of search documents; there is no downward trend in the accuracy of the proposed method.

#### 5.2.2. Returned Document Quantity Effects on Search Time and Accuracy

Next, we tested the search time and search accuracy, whenever a user requested that 10, 20, 30, 40, or 50 documents be returned. We used a set of 500 documents, each varying between 1 KB and 50 KB, with a dictionary ***D_w_*** = 9000, which had a search request that consisted of 10 keywords, where each keyword was assigned a value from 1 to 3.

**Figure 3:**
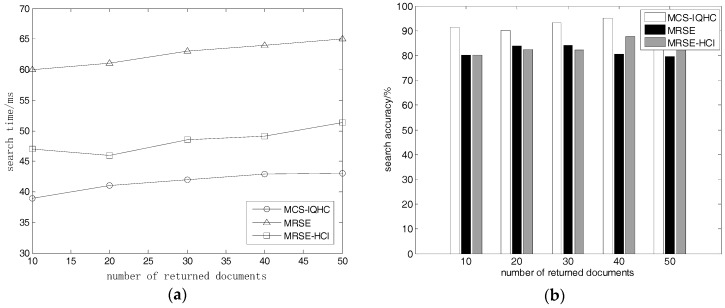
(**a**) Relationship between quantity of returned documents and search time; (**b**) Relationship between quantity of returned documents and search accuracy.

As shown in Figure 3a,b, the search time of MCS-IQHC is significantly lower than the other two methods that we tested, whilst the search accuracy is much higher than others. In the ciphertext search, we compared the similarity between the trapdoor and each cluster center when using MCS-IQHC; the most similar cluster was identified after calculating the similarity between the trapdoor and the cluster in each document, and by choosing the most similar *k* documents. Therefore, the search time and search accuracy were unaffected by any increase in the number of requested documents (*k*) or change in the quantity of returned documents.

#### 5.2.3. Keyword Quantity Effects on Search Time and Accuracy

For the next experiment, we selected 500 documents all varying within 1 KB to 50 KB. In this case, the dictionary size was ***D_w_*** = 9,000, and the user requested the return of 10 documents. We tested the search time and accuracy, when the user’s request consisted of 1, 2, 3, 4, and 5 keywords, respectively.

**Figure 4:**
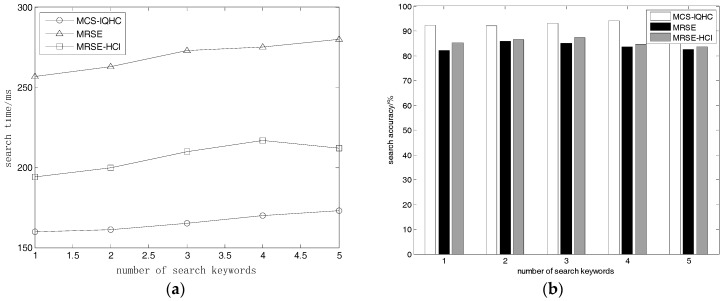
(**a**) Relationship between number of keywords and search time; (**b**) Relationship between number of keywords and search accuracy.

As shown in Figure 4a, MCS-IQHC requires a much lower search time than the other two methods under the same experimental conditions, but the accuracy of MCS-IQHC is also higher as shown in Figure 4b. MRSE search time remained stable; as the number of keywords increased both the search time of MRSE-HCI and MCS-IQHC increased linearly. MCS-IQHC could assign a value to the corresponding position of each keyword when building a search request, based on the number of search keywords. As the keyword quantity increased, the vector dimension of the trapdoor was not reduced, but the search calculation was enhanced; thus, the search time increased linearly. In MCS-IQHC, TF-IDF is combined with VSM to construct a document vector; and the user defined keyword values by demand in the search phase. Documents with important keywords were prioritized for the sake of accuracy, and the search accuracy grew as the number of keywords increased.

## 6. Conclusions

This paper proposed a multi-keyword ciphertext search method for encrypted cloud data, based on the hierarchical clustering index for sensor networks. Document vectors were first constructed through a combination of TF-IDF and VSM, which effectively accounted for the frequency and importance of each keyword in the document set. IQHC was then applied to cluster the document vectors, construct a cluster index and document index, and encrypt them for enhanced ciphertext search efficiency and security. The adaptive ability of the method and the accuracy of the results were continually improved, based on the values of user-customized keywords. The results of a comparative experiment demonstrated that the proposed method was superior to MRSE and MRSE-HCI, in terms of search efficiency, privacy protection, and accuracy.

In our work, the security analysis of the MCS-IQHC was solely based on the Known Ciphertext Model and the Known Background Knowledge Model. In the multi-keyword ciphertext search in the cloud environment, there may be more forms of attacks against user privacy. Furthermore, MCS-IQHC does not consider the dynamic update function of data. There may be operations such as supplementing, modifying, and deleting data uploaded by the data owner to the cloud server. Therefore, in the next step of research, in terms of safety analysis, this method can be further studied and analyzed to improve the ability to dynamically update data.
